# TRPA1-Mediated Src Family Kinases Activity Facilitates Cortical Spreading Depression Susceptibility and Trigeminovascular System Sensitization

**DOI:** 10.3390/ijms222212273

**Published:** 2021-11-12

**Authors:** Lingdi Nie, Liwen Jiang, John P. Quinn, Blair D. Grubb, Minyan Wang

**Affiliations:** 1 Centre for Neuroscience, Department of Biological Sciences, Xi’an Jiaotong-Liverpool University (XJTLU), Suzhou 215123, China; lingdi.nie@xjtlu.edu.cn (L.N.); Liwen.jiang@xjtlu.edu.cn (L.J.); 2 Department of Pharmacology and Therapeutics, Institute of Systems, Molecular and Integrative Biology, Liverpool L69 7ZB, UK; jquinn@liverpool.ac.uk (J.P.Q.); b.d.grubb@dundee.ac.uk (B.D.G.)

**Keywords:** migraine, transient receptor potential ankyrin 1, Src family kinases, cortical spreading depression, trigeminovascular system, protein kinase A, calcitonin gene-related peptide, IL-1β, neuroinflammation, trigeminal ganglia

## Abstract

Transient receptor potential ankyrin 1 (TRPA1) plays a role in migraine and is proposed as a promising target for migraine therapy. However, TRPA1-induced signaling in migraine pathogenesis is poorly understood. In this study, we explored the hypothesis that Src family kinases (SFKs) transmit TRPA1 signaling in regulating cortical spreading depression (CSD), calcitonin gene-related peptide (CGRP) release and neuroinflammation. CSD was monitored in mouse brain slices via intrinsic optical imaging, and in rats using electrophysiology. CGRP level and IL-1β gene expression in mouse trigeminal ganglia (TG) was detected using Enzyme-linked Immunosorbent Assay and Quantitative Polymerase Chain Reaction respectively. The results showed a SFKs activator, pYEEI (EPQY(PO3H2)EEEIPIYL), reversed the reduced cortical susceptibility to CSD by an anti-TRPA1 antibody in mouse brain slices. Additionally, the increased cytosolic phosphorylated SFKs at Y416 induced by CSD in rat ipsilateral cerebral cortices was attenuated by pretreatment of the anti-TRPA1 antibody perfused into contralateral ventricles. In mouse TG, a SFKs inhibitor, saracatinib, restored the CGRP release and IL-1β mRNA level increased by a TRPA1 activator, umbellulone. Moreover, umbellulone promoted SFKs phosphorylation, which was reduced by a PKA inhibitor, PKI (14–22) Amide. These data reveal a novel mechanism of migraine pathogenesis by which TRPA1 transmits signaling to SFKs via PKA facilitating CSD susceptibility and trigeminovascular system sensitization.

## 1. Introduction

Migraine is a disabling neurovascular disorder characterized by a unilateral throbbing headache and multiple neurological symptoms including hypersensitivity to light, sound and smell [1]. Migraine is also a multifactorial disease attributed to both environmental [2] and genetic factors [3]. Migraine pathogenesis is widely accepted to be attributed to neuroinflammation [4,5], sensitization of the trigeminovascular system (TVS) [6] and cortical spreading depression (CSD) [7], a propagating wave on cerebral cortex resulting from temporary excitation followed by depression of neurons and glial cells [7,8]. CSD elicits redistribution of multiple ions, release of neurotransmitters, activation of membrane receptors and neuroinflammation, leading to pathological events including activation of meningeal nociceptors [7,8]. Meningeal nociceptors originate in trigeminal ganglia (TG) and their activation ensues sensitization of the TVS [9]. In this pathway, TG are a pivotal intersection connecting periphery and central regions morphologically and functionally. Being enriched with neuropeptides and inflammatory mediators, active intraganglionic signaling occurs within TG, contributing to peripheral and central sensitization [10]. Currently-used drugs specific for migraine treatment are mainly triptans targeting 5-HT receptors, gepants targeting calcitonin gene-related peptide (CGRP) receptors and CGRP monoclonal antibodies [11,12,13,14]. The development of these drugs has significantly advanced migraine therapy, yet low response rate exists and long-term toxicity is not yet certain. Efforts are still needed to develop novel drugs with higher efficacy and tolerability.

Transient receptor potential ankyrin 1 (TRPA1) has drawn increasing attention to play a role in migraine pathogenesis [15,16,17,18,19]. TRPA1 can be activated by a variety of exogenous and endogenous irritants, some of which are migraine triggers in people with migraine, including umbellulone (UMB) and nitric oxide (NO) [16]. TRPA1 plays an important part in the TVS and peripheral sensitization. In particular, intranasal or dural application of TRPA1 agonists increases meningeal blood flow, neurogenic inflammation, nociceptive behaviors, intraganglionic transmission and release of a known target for migraine prevention and therapy, CGRP [15,20,21,22]. These effects can be mediated by TRPA1 via pharmacological and genetic inhibition of TRPA1. Notably, CGRP receptor antagonists block the TRPA1 agonists-enhanced meningeal blood flow [20], indicating TRPA1-mediated migraine-like symptoms might be dependent on CGRP. There might be a positive loop between TRPA1 activation and CGRP release in migraine pathogenesis, but which needs clarification. In addition, TRPA1 not only mediates oxidative stress in TG and nociceptive behaviors in a migraine model induced by NO donors [17,23], but also plays a central role in modulating cortical neuronal activity [24] and cortical susceptibility to CSD [18], the latter of which also involves CGRP [19]. Some anti-migraine drugs, including sumatriptan and valproic acid, can attenuate the TRPA1-mediated migraine-related allodynia and meningeal blood flow [21,25]. This evidence underpins TRPA1 as one of the most promising targets for migraine therapy.

Despite the above, how TRPA1 activation initiates an intracellular pathway to modulate migraine pathogenesis is still elusive. The modulatory mode of TRPA1 channel in migraine pathogenesis may involve several intracellular proteins [26], in particular protein kinase A (PKA). PKA is a potent regulator of TRPA1 activity as it can induce TRPA1 trafficking to plasma membrane in nociceptive neurons [27] or mediate bradykinin sensitization of TRPA1 in dorsal root ganglion in an inflammatory pain model [28]. Interestingly, PKA activates Src family kinases (SFKs) in multiple cell types [29] and the PKA/SFKs pathway regulates pain sensitivity [30], indicating that SFKs may be a downstream molecule of TRPA1 pathway in pain conditions. SFKs are a class of nonreceptor protein kinases that are reported to regulate TRPA1-mediated calcium influx in cell models [31]. In fact, SFKs have similar roles to TRPA1 in mediating multiple migraine-associated events including CSD [32], brain perfusion [33], nociceptive behavior, CGRP expression and release [34,35]. Therefore, it is likely that SFKs can transmit TRPA1 signaling, contributing to migraine pathogenesis. In the present study, we explored whether SFKs is a downstream signal of TRPA1 in mediating cortical susceptibility to CSD and in the sensitization of TVS as indicated by CGRP release and IL-1β gene expression in mouse TG. Whether PKA bridges the interaction between TRPA1 and SFKs and whether the latter two proteins couple physically in TG were also investigated.

## 2. Results

### 2.1. Co-Expression of TRPA1 and SFKs in Cerebral Cortices of Mice

**Figure 1:**
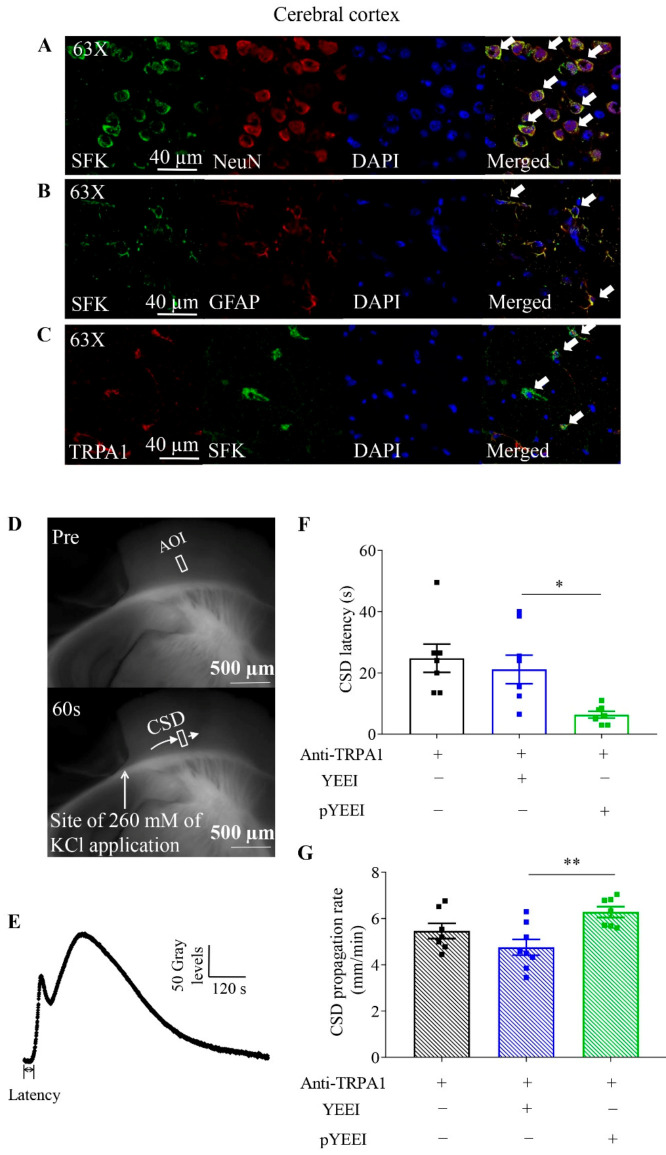
Co-localization of TRPA1 and SFKs, and effects of pYEEI, the SFKs activator, on the anti-TRPA1 antibody-reduced cortical susceptibility to CSD in the mouse brain slice. (**A**,**B**) Representative images of expression of SFKs in neurons and astrocytes of mouse cerebral cortices. (**C**) Representative images of co-localization of TRPA1 and SFKs in mouse cerebral cortices. Staining with DAPI indicated nucleus as shown in blue; staining with the anti-TRPA1 antibody was shown in red; staining with the anti-NeuN antibody indicated neurons and staining with the anti-GFAP antibody indicated astrocytes as shown in green. Double immune-labeling showed expression of SFKs in neurons and astrocytes of mouse cerebral cortices or co-expression of TRPA1 and SFKs in mouse cerebral cortices (white arrows, *n* = 3 per group). (**D**) Representative images of the coronal slice of mouse brain before (upper) and after (lower) CSD induced by ejection of 260 mM KCl in the cerebral cortical region. The same area of interest (AOI) along CSD wave front (pointed by the short arrow) was selected and used for all images. (**E**) The averaged gray level within the AOI was plotted against time to generate the CSD curve. (**F**,**G**) Effects of 0.015 µM anti-TRPA1 antibody (Anti-TRPA1) (*n* = 7), 0.015 µM anti-TRPA1 antibody + 0.1 µM pYEEI (*n* = 7), or 0.015 µM anti-TRPA1 antibody + 0.1 µM YEEI (*n* = 8) on CSD latency (seconds, s) and CSD propagation rate (mm/minute, mm/min) in mouse brain slices. Group data were presented as mean ± SEM. Two-tailed unpaired *t*-test was used for comparison between pYEEI and YEEI group in the presence of anti-TRPA1 antibody. Significance differences were shown as * *p* < 0.05, ** *p* < 0.01.

We detected a co-distribution pattern of TRPA1 and SFKs in cerebral cortices of mice. The results showed that SFKs immunoreactivity was observed in neurons labeled by NeuN (Figure 1A) and astrocytes labeled by GFAP in cerebral cortices of mice (Figure 1B). Additionally, co-expression of TRPA1 and SFKs, largely on plasma membrane, was seen in cerebral cortices of mice (Figure 1C). Immunoreactivity was not seen in the control brain slices stained with respective secondary antibody only (data not shown). These data laid out a foundation for potential physical and/or functional interaction between TRPA1 and SFKs in the mouse cerebral cortex.

### 2.2. pYEEI Restored the Anti-TRPA1 Antibody-Reduced Cortical Susceptibility to CSD in Mouse Brain Slices

Since either TRPA1 or SFKs activity is capable of regulating cortical susceptibility to CSD [12,13,26], we investigated whether they have functional interaction to regulate CSD latency and propagation rate. In this present study, after preincubation of brain slices with the anti-TRPA1 antibody (0.015 µM) that specifically binds to the first extracellular loop of TRPA1 [36] for an hour, the CSD latency and propagation rate were 24.79 ± 4.62 s and 5.46 ± 0.33 mm/minute (*n* = 7 respectively, Figure 1F,G) [18]. These two CSD parameters were unaffected by 0.1 µM YEEI (*n* = 8), the negative control of the SFKs activator, pYEEI (EPQY(PO3H2)EEEIPIYL) and the CSD latency and propagation rate were 21.13 ± 4.67 s and 4.75 ± 0.34 mm/minute, respectively (Figure 1F,G). Interestingly, compared to the data in the YEEI group in the presence of the anti-TRPA1 antibody, 0.1 µM pYEEI (*n* = 7) significantly reduced CSD latency to 6.36 ± 1.13 s (*p* = 0.0157) and increased CSD propagation rate to 6.28 ± 0.23 mm/minute (*p* = 0.0031) (Figure 1F,G). These data support that the reduced cortical susceptibility to CSD by TRPA1 deactivation was functionally reversed by SFKs activation.

### 2.3. The Anti-TRPA1 Antibody Reduced the Level of Phosphorylated SFKs at Y416 Induced by CSD in Cytosols of Rat Cerebral Cortices

**Figure 2:**
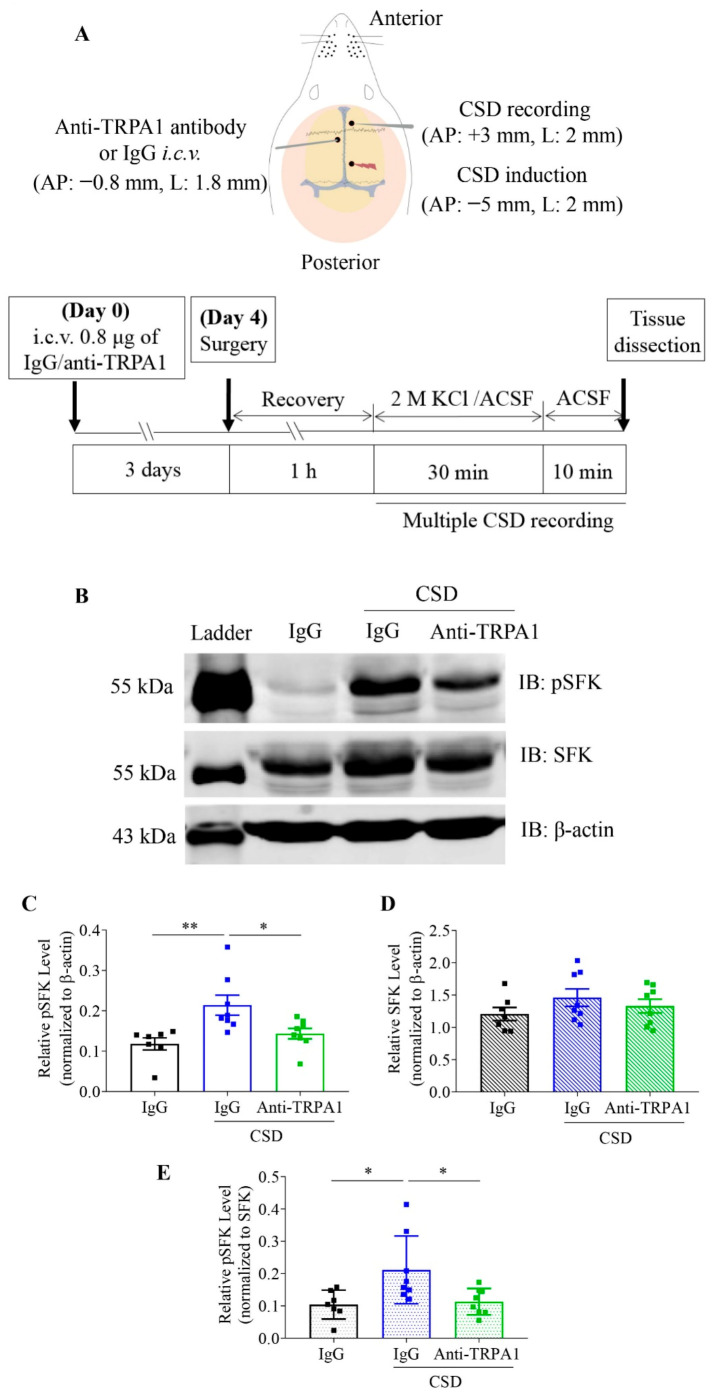
Anti-TRPA1 antibody reduced phosphorylation of SFKs at Y416 induced by multiple CSD in cytosols of rat cerebral cortices. (**A**) Diagram showing brain coordinates for CSD induction, CSD recording and drug perfusion (Upper) as well as the in vivo experimental procedure (lower). (**B**) Representative images showing Western blot analysis of expression of phosphorylated SFKs (pSFKs) at Y416, SFKs and β-actin in cytosolic cerebral cortices of rats treated with IgG or anti-TRPA1 antibody (Anti-TRPA1) at 0.8 μg with or without 2 M K^+^-induced CSD. (**C**,**D**) Data analysis of the levels of phosphorylated SFKs at Y416 or SFKs relative to that of β-actin (absolute ratio in band intensity) and (**E**) comparison of relative pSFK normalized to SFK levels (absolute ratio in band intensity) in cytosolic cerebral cortices of rats treated with IgG without CSD induction (*n* = 7), IgG with CSD induction (*n* = 8) or anti-TRPA1 antibody with CSD induction (*n* = 8). Group data were presented as mean ± SEM. Two-tailed unpaired *t*-test was used for comparison between IgG without CSD and IgG with CSD group, IgG with CSD and anti-TRPA1 antibody with CSD group. Significance differences were shown as * *p* < 0.05, ** *p* < 0.01.

We next investigated if SFKs are downstream of TRPA1 signaling by examining whether multiple CSD-induced elevation of SFKs activity could be regulated by TRPA1. The results showed that multiple CSD significantly augmented phosphorylated SFKs level (absolute ratio in band intensity normalized to β-actin) to 0.21 ± 0.02 (*n* = 8) in cytosols of rat cerebral cortices compared with that at 0.12 ± 0.02 without CSD induction (*n* = 7, *p* = 0.0068, Figure 2C). As expected, perfusion of 0.8 μg of the anti-TRPA1 antibody into the contralateral intracerebral ventricle 4 days before CSD induction significantly decreased the relative phosphorylated SFKs to 0.14 ± 0.01 in ipsilateral cortices of rats (*n* = 8, *p* = 0.0293) when compared with the CSD only group (Figure 2C). Unlike phosphorylated SFKs, there was no significant difference in the level of total SFKs (absolute ratio in band intensity normalized to β-actin) among all groups (Figure 2D). Consistently, when the level of phosphorylated SFKs was normalized to SFKs (absolute ratio in band intensity), a significant increase in phosphorylated SFKs was also observed at 0.21 ± 0.04 in the multiple CSD group when compared with that at 0.10 ± 0.02 in the without CSD group (*p* = 0.0255, Figure 2E). The increase in phosphorylated SFKs by CSD was reduced to 0.11 ± 0.01 by the anti-TRPA1 antibody (*p* = 0.0351, Figure 2E). These data show that multiple CSD-triggered SFKs phosphorylation was due to elevated activation of SFKs but no increase in total SFKs. Collectively, the data suggest the anti-TRPA1 antibody is capable of reducing CSD-induced SFKs activation in ipsilateral cortices of rats.

### 2.4. Co-Localization of TRPA1 and SFKs in Mouse TG

**Figure 3:**
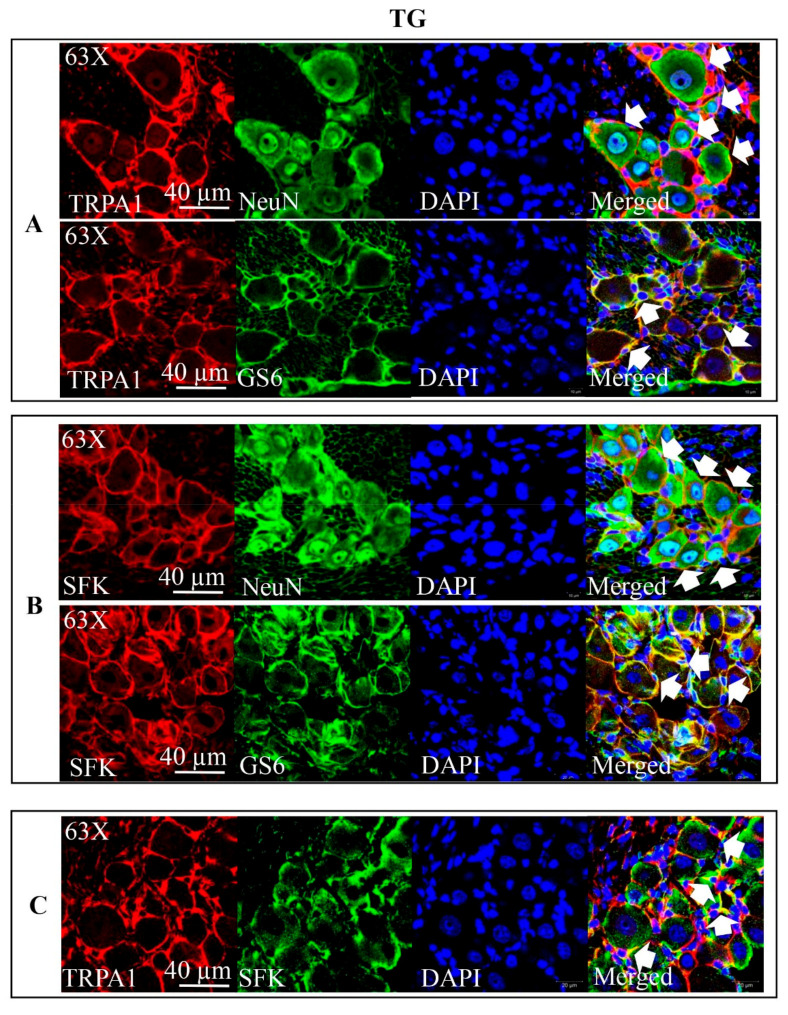
TRPA1 and SFKs were co-localized in mouse TG. (**A**,**B**) Representative images of expression of TRPA1 or SFKs in neurons and satellite glial cells of mouse TG. (**C**) Representative images of co-localization of TRPA1 and SFKs in mouse TG. Staining with DAPI indicated nucleus as shown in blue; staining with anti-TRPA1 antibody was shown in red; staining with anti-NeuN antibody indicated neurons and staining with anti-GS6 antibody indicated satellite glial cells as shown in green. Double immune-labeling with indicated antibodies showed expression of TRPA1 and SFKs in neurons and satellite glial cells of TG or co-expression of TRPA1 and SFKs in TG (white arrows, *n* = 3 per group).

As TRPA1 is also highly involved in the sensitization of the TVS, we next investigated whether SFKs could transmit TRPA1 signaling to mediate the activation and sensitization of the TVS. It is known that TRPA1 expresses in substantial population of trigeminal nociceptors originating in TG and in TG neurons [37,38] and SFKs were also previously identified to exist in TG [39]; here, we further detected respective distribution of TRPA1 or SFKs in neurons and satellite glial cells of mouse TG and their co-localization in mice TG. The results showed that TRPA1 immunoreactivity was present in nearly all-sized TG neurons (*n* = 3 per group, Figure 3A). TRPA1 immunoreactivity was also observed in satellite glial cells labeled with GS6 (*n* = 3 per group, Figure 3A). Similar to TRPA1, the presence of SFKs immunoreactivity was also observed in both TG neurons and satellite glial cells (*n* = 3 per group) (Figure 3B), which extended previous findings that SFKs express in mouse TG by immunoblotting [39]. Additionally, results on double staining with the anti-TRPA1 antibody and anti-SFKs antibody showed their co-localization largely on plasma membrane of TG cells of mice (*n* = 3 per group, Figure 3C).

### 2.5. Saracatinib Reversed CGRP Release and IL-1β mRNA Promoted by Umbellulone in Mouse TG

**Figure 4:**
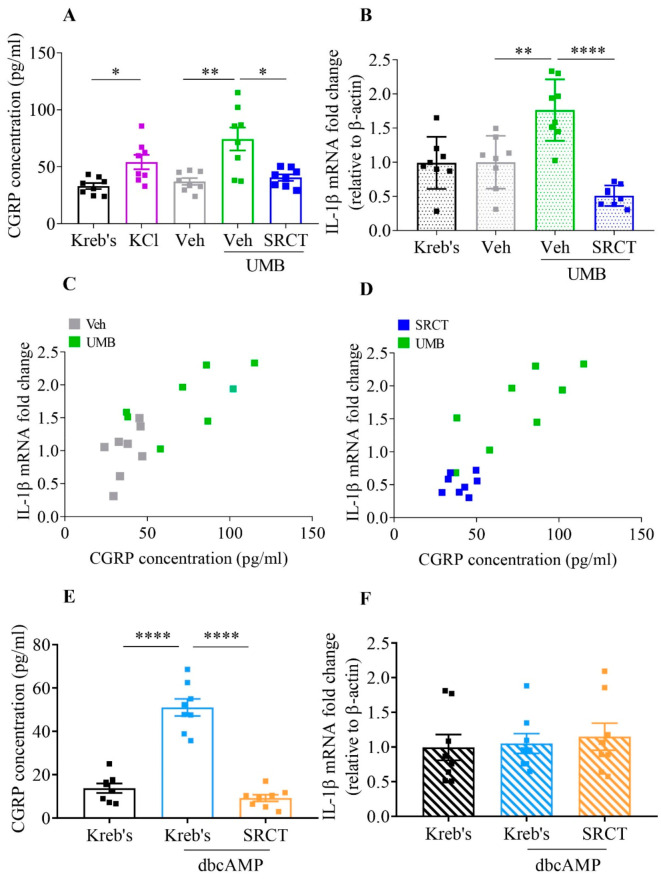
Saracatinib, the SFKs inhibitor, reduced the TRPA1 activator umbellulone-promoted CGRP release and IL-1β mRNA and the PKA activator dbcAMP-promoted CGRP release in the mouse TG. (**A**,**B**) Effects of Kreb’s solution, 40 mM KCl, 0.06% DMSO (Veh), 600 μM umbellulone (UMB) or 600 μM umbellulone + 1.5 μM saracatinib (SRCT) on CGRP release (pg/mL) and IL-1β mRNA fold change relative to β-actin in mouse TG (*n* = 8 per group). (**C**,**D**) Correlation between CGRP release and IL-1β mRNA fold change in mouse TG treated by 0.06% DMSO, 600 μM umbellulone or 600 μM umbellulone + 1.5 μM saracatinib (*n* = 8 per group). (**E**,**F**) Effects of Kreb’s solution, 300 μM dbcAMP or 300 μM dbcAMP + 1.5 μM saracatinib on CGRP release (pg/mL) and IL-1β mRNA fold change relative to β-actin in mouse TG (*n* = 8 per group). Group data were presented as mean ± SEM. Two-tailed unpaired t-test was used for comparison between Kreb’s and KCl group, vehicle and umbellulone group, vehicle and saracatinib group in the presence of umbellulone, Kreb’s and dbcAMP group, Kreb’s and saracatinib group in the presence of dbcAMP. Two-tailed Pearson correlation was used for correlation analysis between CGRP release and IL-1β mRNA fold change in each group. Significance differences were shown as * *p* < 0.05, ** *p* < 0.01, **** *p* < 0.0001.

In order to explore the role of TRPA1/SFKs signaling in the TVS sensitization, we examined whether SFKs activity could mediate TRPA1-triggered CGRP release and IL-1β gene expression in mouse TG [10]. Ex vivo mouse TG was cultured by application of KCl that is known to promote CGRP release from the tissue [40] to confirm the validity of the method. The results showed that incubation of 40 mM KCl for 20 min induced a significant increase in CGRP level in the culture medium to 54.1 ± 6.3 pg/mL when compared to 33.0 ± 2.6 pg/mL in Kreb’s control group (*n* = 8 per group, *p* = 0.0122) (Figure 4A). Consistent to a previous finding [15], 600 μM umbellulone markedly triggered TG to release CGRP at 74.3 ± 10.1 pg/mL, while the CGRP level in the 0.06% DMSO group was 37 ± 3 pg/mL (*n* = 8 per group, *p* = 0.0071) (Figure 4A). As expected, 1.5 μM saracatinib significantly reversed the level of CGRP triggered by 600 μM umbellulone to 40.5 ± 2.8 pg/mL (*n* = 8 per group, *p* = 0.0118) (Figure 4A).

In terms of IL-1β gene expression, the vehicle, 0.06% DMSO, did not alter IL-1β mRNA level compared with the Kreb’s group (*n* = 8 per group, Figure 4B). Consistent to CGRP release, 600 μM umbellulone induced TG IL-1β gene expression and the mRNA level was 1.8 ± 0.2 (*n* = 8, fold change) in comparison with that at 1 ± 0.1 in the vehicle group (*n* = 8, *p* = 0.0028, Figure 4B). The induction of IL-1β gene expression induced by TRPA1 activation was markedly decreased by 1.5 μM saracatinib to 0.5 ± 0.1 (*n* = 8) (*p* < 0.0001, Figure 4B). Correlation analysis showed a positive relationship between the increased levels of CGRP and IL-1β mRNA in TG treated by umbellulone (r = 0.7726, *p* = 0.0005) (Figure 4C). Such a positive correlation was also confirmed between the reversed CGRP release and IL-1β mRNA level in TG when umbellulone and saracatinib were co-applied (r = 0.8521, *p* < 0.0001) (Figure 4D).

### 2.6. Saracatinib Reduced CGRP Release Promoted by Dibutyryl-cAMP (dbcAMP), Which Is Ineffective on IL-1β mRNA Level, in Mouse TG

As PKA is able to sensitize TRPA1 and activate SFKs in multiple cell types [29,41], we examined if PKA is involved in the TRPA1-dependent SFKs signaling in regulating CGRP release and IL-1β gene expression in TG. Similar to umbellulone (Figure 4A), 300 μM dbcAMP significantly promoted the level of CGRP in the media to 51.04 ± 3.93 pg/mL compared to that in Kreb’s control group (*n* = 8 per group, *p* < 0.0001). This increase in CGRP level was reduced by 1.5 μM saracatinib to 9.22 pg/mL (*n* = 8 per group, *p* < 0.0001) (Figure 4E). Unlike umbellulone, 300 μM dbcAMP did not alter IL-1β mRNA level as compared to that in Kreb’s control group (1.05 ± 0.14 vs. 0.99 ± 0.19, *n* = 8 per group, *p* = 0.8145), nor there was any change in IL-1β mRNA level by saracatinib (Figure 4F).

### 2.7. PKI (14–22) Amide Reduced the Level of Phosphorylated SFKs at Y416 Promoted by Umbellulone in Mouse TG

**Figure 5:**
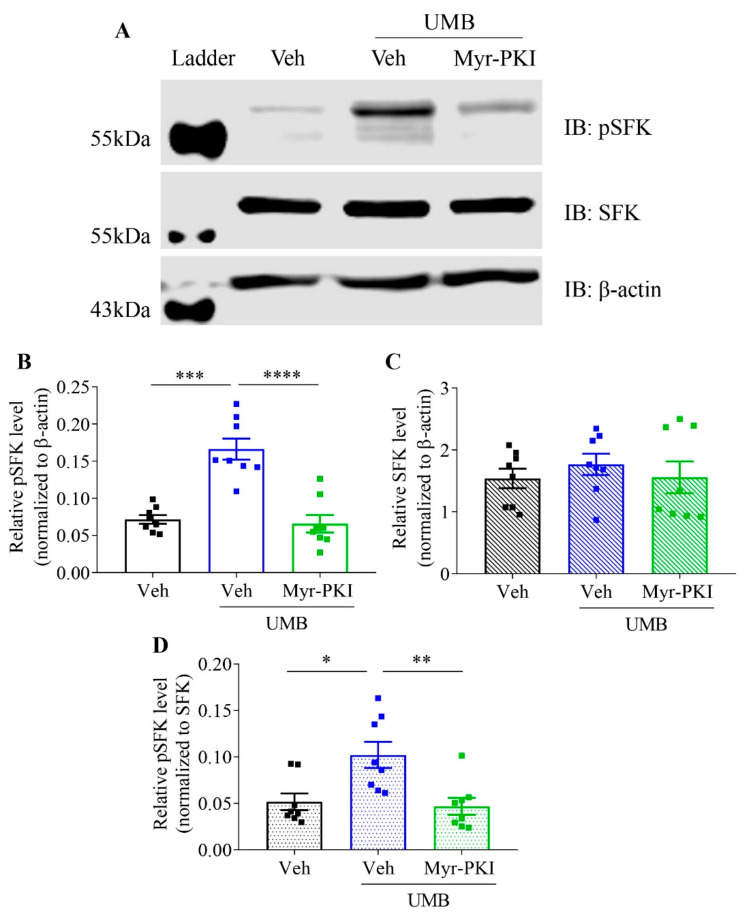
Umbellulone, the TRPA1 activator, promoted phosphorylation of SFKs at Y416, which was reduced by PKI (14–22) Amide, the PKA inhibitor, in the mouse TG. (**A**) Representative images showing Western blot analysis of expression of phosphorylated SFKs (pSFKs) at Y416, SFKs and β-actin in mouse TG treated by 0.06% DMSO (Veh), 600 μM umbellulone (UMB) or 600 μM umbellulone + 10 μM PKI (14–22) Amide (Myr-PKI); (**B**,**C**) data analysis of the expression levels of phosphorylated SFKs at Y1416 or SFKs relative to that of β-actin (absolute ratio in band intensity) and (**D**) comparison of relative pSFK/SFK levels (absolute ratio in band intensity) in mouse TG treated by 0.06% DMSO, 600 μM umbellulone or 600 μM umbellulone + 10 μM PKI (14–22) Amide (*n* = 8 per group). Group data were presented in as mean ± SEM. Two-tailed unpaired *t*-test was used for comparison between vehicle and umbellulone group, vehicle and PKI (14–22) Amide group in the presence of umbellulone. Significance differences were shown as * *p* < 0.05, ** *p* < 0.01, *** *p* < 0.001, or **** *p* < 0.0001.

We predicted that PKA could mediate TRPA1-induced SFKs phosphorylation in TG. To test this hypothesis, we first examined whether TRPA1 could trigger SFKs activation in TG. Indeed, when the level of phosphorylated SFKs was normalized to β-actin (absolute ratio in band intensity), 600 μM umbellulone significantly increased level of phosphorylated SFKs at Y416 to 0.17 ± 0.01, compared to that at 0.07 ± 0.01 in the vehicle group (*n* = 8 per group, *p* < 0.0001) (Figure 5B). We next examined if PKA inhibition could reduce TRPA1-induced SFKs phosphorylation in TG. The results showed that 10 μM PKI (14–22) Amide significantly reduced the level of phosphorylated SFKs at Y416 to 0.07 ± 0.01 in the presence of umbellulone in comparison with that in umbellulone only group (*n* = 8 per group, *p* = 0.0003) (Figure 5B). In contrast to phosphorylated SFKs, the consistent level of total SFKs in all groups confirmed the total level of SFKs was not modulated by TRPA1 activation, nor by PKA deactivation in the presence of umbellulone (Figure 5C). We also presented the relative level of phosphorylated SFKs normalized to SFKs in absolute ratio in band intensity. The results showed a significant increase in phosphorylated SFKs by umbellulone (0.10 ± 0.01) when compared with the vehicle group (0.05 ± 0.01) (*p* = 0.0107, Figure 5D). This increase was reversed by PKI (14–22) Amide at 0.05 ± 0.01 (*p* = 0.0062, Figure 5D).

### 2.8. TRPA1 Did Not Form a Complex with SFKs in Rat Cerebral Cortices after CSD

**Figure 6:**
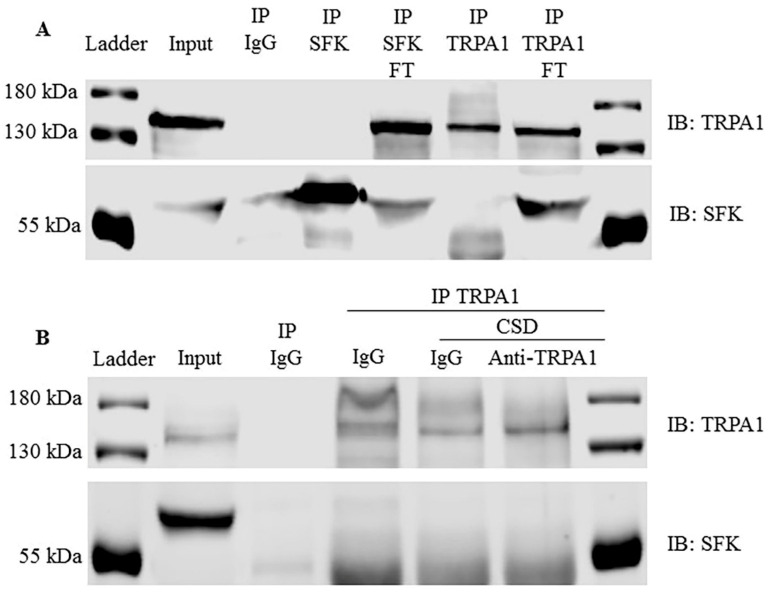
CSD was unlikely to promote the physical interaction between TRPA1 and SFKs in rat cerebral cortices. (**A**) Co-immunoprecipitation between TRPA1 and SFKs in untreated rat cerebral cortices. Either TRPA1 or SFKs was immunoprecipitated (IP) followed by immunoblotting (IB) of the pull-down of TRPA1/SFKs with SFKs/TRPA1. FT: flow-through during IP. (**B**) Co-immunoprecipitation between TRPA1 and SFKs in rat cerebral cortices treated with IgG or anti-TRPA1 antibody (Anti-TRPA1) at 0.8 μg with or without 2 M K^+^-induced CSD. TRPA1 was immunoprecipitated followed by immunoblotting of the pull-down of TRPA1 with SFKs. IgG was used for pull-down in IP as a negative control in (**A**,**B**).

Since SFKs are known to physically interact with multiple membrane proteins such as NMDA receptors to regulate their activities and regulate CSD [42,43], we investigated the possibility of TRPA1 physically interacting with SFKs in untreated rat cerebral cortices. The results showed that neither the anti-TRPA1 antibody nor the anti-SFKs antibody pulled down the other protein (Figure 6A). Similarly, the anti-TRPA1 antibody did not pull down SFKs in rat cerebral cortices treated with 0.8 μg of IgG or anti-TRPA1 antibody after CSD (Figure 6B). These data suggest that TRPA1 and SFKs are unlikely to physically couple with each other in normal conditions and that CSD does not induce their physical interaction.

## 3. Discussion

These findings reveal a novel mechanism of migraine that TRPA1 transmits signaling to SFKs via PKA, which promotes CSD susceptibility and TVS sensitization, leading to migraine progression.

One key finding is that TRPA1-mediated intracellular SFKs activity contributes to CSD propagation. First, there is co-localization of SFKs and TRPA1 in mouse cerebral cortices, either of which proteins are also expressed in mouse cortical neurons and astrocytes (Figure 1A–C). These lay out the foundation for their possible functional relationship. Second, activation of SFKs is capable of reversing the prolonged CSD latency and the reduced CSD propagation rate by pretreatment of the anti-TRPA1 antibody in mouse brain slices (Figure 1F,G). These data support that there is a functional interaction between SFKs activity and TRPA1 channel in contributing to CSD propagation. This evidence is in line with our earlier findings that either TRPA1 or SFKs activity is critical for CSD propagation both in mouse brain slices and in rats [18,32]. The fact that pretreatment of the anti-TRPA1 antibody attenuates multiple CSD-induced SFKs activity Y416 site in cytosols of rat ipsilateral cerebral cortices (Figure 2C) further implies that intracellular SFKs activity is capable of transmitting TRPA1 signaling in cerebral cortices and reinforces the regulatory role of the TRPA1/SFKs pathway in CSD propagation.

Apart from CSD, the present data also reveal that SFKs are crucial for TRPA1 signaling in facilitating nociceptive transmission. Co-distribution of TRPA1 and SFKs are seen largely on plasma membrane of mice TG, either of which proteins are also expressed in TG neurons and satellite glial cells (Figure 3), which renders their functional relationship in TG likely. TRPA1 plays an important role in the TVS [15] in which TG sensitization is a crucial process to facilitate nociceptive transmission and sustaining pain [44]. In TG, deactivation of SFKs is capable of reversing the TRPA1-promoted CGRP release and IL-1β gene expression (Figure 4A,B) and their CGRP and IL-1β gene expression levels are positively correlated (Figure 4C,D). These data support that the functional interaction between TRPA1 and SFKs also exists for TVS sensitization. The fact that TRPA1 activation by umbellulone increases SFKs activity in TG (Figure 5B), confirming that SFKs are activated downstream of TRPA1. On one hand, SFKs regulate CGRP release enhanced by nerve growth factor in dorsal root ganglia neurons [35] and promote cytokine expression in macrophages and microglia [45,46]. On the other hand, the released CGRP from TG neurons provokes release and expression of cytokines in satellite glial cells [47,48], which in turn can augment expression and release of CGRP from neurons [49,50], forming crosstalk signaling for sensitizing the TVS in migraine. Thus, it is likely that the synergistic effects of neuropeptides and cytokines on TVS sensitization are regulated by the TRPA1/SFKs pathway. It is noted that relationship between TRPA1/SFKs pathway in mediating CSD propagation in cerebral cortex and in facilitating TVS sensitization found in TG tissue is still unclear. Given that CSD is known to activate and sensitize the TVS [51,52] and taken together with our above findings, it is possible that the reduced cortical susceptibility to CSD under inhibition of TRPA1/SFKs signaling may subsequently have a negative impact on the hypersensitivity of the TVS induced by CSD or other migraine triggers, but this requires future clarification. 

Although the molecular mechanism underlying how SFKs transmit signaling from TRPA1 in migraine is not fully known, here we identify that PKA serves as an intermediate molecule, transmitting signaling from TRPA1 to SFKs facilitating TVS sensitization. In TG, inhibition of PKA activity reduces SFKs activation triggered by the TRPA1 activator (Figure 5B). Interestingly, PKA activation promotes CGRP release, which is reduced by deactivation of SFKs (Figure 4E). Concordantly, an earlier study shows that PKA regulates LPS-induced CGRP release in dorsal root ganglion (DRG) neurons [53]. These data suggest that PKA directs SFKs to mediate CGRP release in TG. As PKA activation promotes CGRP release but not increases in IL-1β gene expression (Figure 4F), we therefore propose that PKA may direct SFKs to mediate CGRP release but not IL-1β gene expression downstream TRPA1 in migraine. PKA has been identified as a potent regulator of TRPA1 activity via modulating its phosphorylation and membrane trafficking [21,22,35,48]. More importantly, PKA is required for TRPA1-mediated nociception [27] and is also known to activate SFKs in various cell models [29], spinal dorsal horn [30] and hypothalamic arcuate nucleus neurons [54]. Similar to the TRPA1/PKA pathway, the PKA/SFKs pathway regulates pain sensitivity [30]. It is likely that they share the same pathway in regulating pain. Moreover, the PKA/SFKs pathway modulates phosphorylation and synaptic concentration of N-methyl-D-aspartate (NMDA) receptors in spinal dorsal horn and insular cortex [30,55], which is critical for central sensitization. Interestingly, TRPA1 has been found to interact with NMDA receptors physically in the cerebral cortex, spinal cord, DRG tissues [56] and functionally in regulating nociceptive behavior and synaptic transmission [57,58,59]. Therefore, we propose that the TRPA1/PKA/SFKs pathway may exist to play a regulatory role in migraine pathogenesis, possibly involving interaction with NMDA receptors. Since activation of TRPA1 increases calcium influx and PKA and SFKs have rich crosstalk with calcium [60,61], calcium ions might be involved in mediating the signaling among TRPA1, PKA and SFKs. An alternative explanation that may underlie SFKs transmit signaling from TRPA1 in migraine involves protein kinase C (PKC) as this enzyme regulates TRPA1 activity and SFKs activity [48,49,57] and PKC plays a part in TRPA1-mediated nociception [62]. Whether a PKC/SFKs pathway is involved in nociception is worth future investigation.

Thus far, there is no direct evidence of SFKs-mediated phosphorylation of TRPA1, albeit a few putative phosphorylation sites of TRPA1 (tyrosines Y22, Y97 and Y680) by SFKs which are proposed based on prediction servers [26]. Indeed, in the present study, there is no physical interaction between TRPA1 and SFKs in rat cerebral cortices in both the normal condition and immediately after CSD (Figure 6). Additionally, TRPA1 deactivation is unable to reduce CSD-induced SFKs phosphorylation level in membranes of rat cerebral cortices (Supplementary Figure S1). Taken together, SFKs-transmitted TRPA1 signaling is least likely to occur via their direct interaction in migraine pathogenesis. It should be noted that the increased activation level of SFKs on plasma membranes by CSD under this study may be explained by the fact that CSD propagation requires many membrane proteins including NMDA receptors, P2X7 receptors and Pannexin-1, all of which have physical interaction with SFKs [63,64,65].

**Figure 7:**
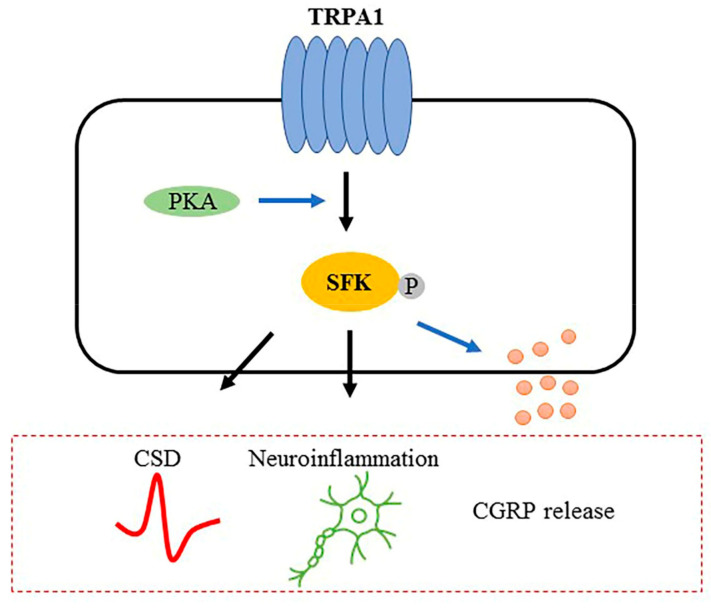
Schematic representation of the proposed TRPA1/SFKs signaling-mediated migraine pathogenesis. TRPA1/SFKs pathway involving PKA contributes to migraine pathogenesis from multiple aspects, including cortical susceptibility to CSD, CGRP release and neuroinflammation.

Nonetheless, one limitation in this study is that only male rodents are used. Since migraine is a prevalent condition on women [66], future work conducted in females is needed to provide better clinical implication for both genders. Another limitation is the extrapolation of the protein level of IL-1β that is likely to be altered by TRPA1-mediated SFKs signaling, especially because IL-1beta mRNA expression and protein expression are well correlated [67]. Thus, we predict that TRPA1-mediated SFKs activation may promote IL-1β protein expression, which, together with the increased CGRP release observed in this study, forms synergistic effects on TVS sensitization to enhance migraine pain transmission. Taken together, our data unravel for the first time that TRPA1-meidated Src Family Kinases activity facilitates CSD susceptibility and trigeminovascular system sensitization (Figure 7), thereby contributing to migraine progression. Whilst it is challenging to translate these research findings from rodents to humans, our study offer mechanistic evidence that potential therapeutic approaches targeting this pathway may provide a new clue for migraine prophylaxis and treatment.

## 4. Materials and Methods

### 4.1. Animals

A total of 34 adult male Sprague-Dawley rats (251.2 ± 6.8 g) and 128 adult male C57BL/6J mice (20.5 ± 0.2 g) were purchased from Shanghai SLAC Laboratory Animal Corporation Ltd. (Shanghai, China) Only male mice/rats were used in this study in order to minimize possible impact of sex hormones as TRPA1 channel activity is regulated by catechol estrogens in pancreatic β-cells [68]. Animals were housed in the Experimental Animal Center of Soochow University for at least one week to be acclimated to the housing room before use. Animal procedures were approved by the Research Ethical Committee of Xi’an Jiaotong-Liverpool University (XJTLU, code: EXT 19-01-04, approved on 4 March 2019) under the agreement with Soochow University and performed in accordance with relevant national and provincial guidelines. Random experimental groups were performed per day and all animals were randomly allocated into different experimental groups.

### 4.2. Immunohistochemistry

We first detected whether TRPA1 and SFK co-express in the mouse cerebral cortex and TG, respectively. Brain or TG slices of mice (*n* = 3) were prepared as described earlier [19]. Coronal sections (20 μm) were prepared between 1 to 1.5 mm posterior to brain bregma, or in neuron-enriched regions of TG, using a cryostat (CM1950 Leica, Wetzlar, Germany). Slices were incubated in 10% goat serum (AR0009 Boster Biological Technology, Pleasanton, CA, USA) at room temperature (RT) for 2 h. Brain slices were incubated in anti-SFKs antibody (1:80, AF3389 R&D Systems, Minneapolis, MN, USA) with anti-NeuN antibody (1:500, MAB377 Merck, St. Louis, MO, USA), or anti-GFAP antibody (1:100, 3670 CST, Beverly, MA, USA) or anti-TRPA1 antibody (1:100, ACC-037 Alomone Labs, Jerusalem, Israel), respectively at 4 °C overnight. TG slices were incubated in the following antibodies at 4 °C overnight: anti-SFKs antibody (1:200, 2109 CST, Beverly, MA, USA) with either the anti-NeuN antibody or anti-GS6 antibody (1:100, MAB302, Merck, St. Louis, MO, USA); the anti-SFKs antibody (1:20) with the anti-TRPA1 antibody (1:100) that binds to the first extracellular loop of membrane TRPA1 [36]. Next, all slices were incubated in Alexa fluor 568 goat anti-rabbit secondary antibody (1:300, A11036 Invitrogen, Carlsbad, CA, USA) with 488 goat anti-mouse secondary antibody (1:300, A11029 Invitrogen, Carlsbad, CA, USA) or 488 donkey anti-goat secondary antibody (1:300, A11055 Invitrogen, Carlsbad, CA, USA) at RT for 1 h, followed by incubating in 4′,6-diamidino-2-phenylindole (DAPI) (1:5000, D8417 Sigma-Aldrich, St. Louis, MO, USA) at RT for 5 min. After mounting the slices with mounting solution (S36936 Invitrogen, Carlsbad, CA, USA), targeted proteins in cells were imaged using a confocal laser-scanning microscope (LSM880 Zeiss, Jena, Germany).

### 4.3. CSD Induction and Recording

#### 4.3.1. Induction and Recording of CSD by Intrinsic Optical Imaging in Mouse Brain Slices and Experimental Design

Preparation of mouse brain slices and intrinsic optical imaging of CSD in mouse brain slices were performed as described previously [65]. Briefly, CSD was induced by ejection of 260 mM KCl in layer 3 to 5 of each coronal section of cerebral cortex (thickness of 400 µm). Intrinsic optical images were recorded and digitized using a camera (ROL-XR-F-M-12, Qimaging, Media Cybernetics, Marlow, UK) and Image Pro Plus software. The effect of a SFKs activator, pYEEI (A^+^ Peptide, Shanghai, China), or its negative control, YEEI (sequence: EPQYEEEIPIYL, A^+^ Peptide, Shanghai, China), on CSD latency and propagation rate in the presence of the above-stated anti-TRPA1 antibody were examined. Three groups were designed: (i) 0.015 µM anti-TRPA1 antibody (*n* = 7); (ii) 0.015 µM anti-TRPA1 antibody + 0.1 µM pYEEI (*n* = 7); (iii) 0.015 µM anti-TRPA1 antibody + 0.1 µM YEEI (*n* = 8). Each brain slice was incubated with anti-TRPA1 antibody for 1 h and then with pYEEI or YEEI for another 1 h. CSD induction and recording was carried out 15 min before stopping application of pYEEI or YEEI. A biphasic curve was generated by plotting the average gray level within a selected area of interest against time for analysis of CSD. CSD latency (the time interval between KCl ejection and CSD elicitation) and propagation rate were calculated to reflect cortical susceptibility to CSD.

#### 4.3.2. Induction and Recording of CSD by Electrophysiology in Rats and Experimental Design

Induction and recording of CSD by electrophysiology in rats pretreated with the anti-TRPA1 antibody were performed as described in details in the above and in our previous publication [19]. Briefly, rats were anesthetized using isoflurane (5% for induction, 2–3% for surgery, 1–2% for maintenance) in O_2_ and N_2_O (1:2), and a burr hole was drilled on the left skull for cannula implantation into the intracerebral ventricle, followed by perfusion of 0.8 μg of the anti-TRPA1 antibody or IgG (D110502, BBI Life Sciences, Shanghai, China), the latter of which was applied as a negative control of the anti-TRPA1 antibody. Four days after drug perfusion, another two burr holes on the right skull were drilled under anesthesia for CSD induction (dura intact) and recording via an Ag/AgCl electrode, followed by 1 h stabilization with continuous perfusion of artificial cerebral spinal fluid. We previously showed that a single CSD induces activation of SFKs in rat ipsilateral cerebral cortices via increasing the level of phosphorylated SFKs at Y416 [32]. As both single and multiple CSD events are associated with migraine, in the present study, multiple CSD waves were induced by continuous perfusion of 2 M KCl into the posterior hole for 30 min [19]. Electrophysiological devices (Digitimer, Welwyn Garden City, UK) and Labview (National Instrument, Austin, TX, USA) were used to digitize and record CSD. EEG was monitored to indicate the depth of anesthesia. In order to examine whether the anti-TRPA1 antibody was capable of reducing the level of ipsilateral phosphorylated SFKs induced by CSD, three groups were designed: (i) 0.8 μg of IgG without CSD induction as sham control (*n* = 7); (ii) 0.8 μg of IgG with CSD induction (*n* = 8); (iii) 0.8 μg of the anti-TRPA1 antibody with CSD induction (*n* = 8). After recording CSD, rats were sacrificed immediately using 5% isoflurane in N_2_O, and ipsilateral cerebral cortices were dissected for subsequent cytosolic and membrane protein fractionation to detect the level of cytosolic and membrane phosphorylated SFKs at Y416 by Western blot (WB).

### 4.4. Protein Expression Analysis

#### 4.4.1. Protein Extraction

Total proteins were extracted by homogenizing the brain tissue in 2% SDS (74255 Sigma-Aldrich, St. Louis, MO, USA)) (for Western blot) or 1% NP40 (26146 Thermo Scientific, Waltham, MA, USA) (for co-immunoprecipitation) with protease inhibitor (04693116001 Roche, Indianapolis, IN, USA) and phosphatase inhibitor (5870 CST, Beverly, MA, USA). Protein concentration was measured using a Bicinchoninic Acid Protein Assay Kit (P0010 Beyotime, Shanghai, China). Cytosolic and membrane proteins of rat cerebral cortices were separated using a Membrane and Cytosol Protein Extraction Kit (P0033 Beyotime, Shanghai, China).

#### 4.4.2. Western Blot

Protein expression level was analyzed by WB. Protein samples were denatured with SDS-PAGE sample loading buffer (P0015 Beyotime, Shanghai, China) at 100 °C for 5 min. The protein samples were separated on a 10% sodium dodecyl sulfate polyacrylamide gel and then transferred onto nitrocellulose (NC) membranes (66485 Pall, Pensacola, FL, USA). The NC membranes were incubated in 5% milk at RT for 1 h, followed by incubating in anti-phospho-Y416 SFKs antibody (1:500 or 1:200, 6943 CST, Beverly, MA, USA) and anti-β-actin antibody (1:2000, 4970 CST, Beverly, MA, USA) at 4 °C overnight. Subsequently, the NC membranes were incubated in IRDye 680RD donkey anti-rabbit secondary antibody (1:5000, 925-68073 LI-COR, Lincoln, NE, USA) at RT for 1 h. An Odyssey Near-Infrared Fluorescent Imaging System (LI-COR, Lincoln, NE, USA) was used to detect fluorescent signals at 700 nm, which represented targeted proteins, on the membranes. Next, the anti-phospho-Y416 SFKs antibody on the membranes was stripped off using 0.2 M NaOH (134070010 Acros Organics, Geel, Belgium) at RT for 15 min for staining with anti-SFKs antibody (1:1000, 2109 CST, Beverly, MA, USA). The same process as above was repeated for imaging the band intensity of SFKs. The expression levels of targeted proteins were quantified using Image Studio Lite software. For each sample, the mean gray value of each band was calculated and the level of each phosphorylated SFKs was subsequently presented as absolute ratio in band intensity between pSFKs and β-actin, or SFKs and β-actin, or pSFKs and SFKs.

### 4.5. Mouse TG Tissue Culture and Experimental Design

Mice were sacrificed by rapid cervical dislocation. Both the left and right TG of one mouse were collected and used as one experiment. These TG were incubated in 300 μL of preoxygenated Kreb’s solution (composition in mM: 126 NaCl, 2.5 KCl, 2.4 CaCl_2_•2H_2_O, 1.3 MgCl_2_•6H_2_O, 18 NaHCO_3_, 1.2 NaH_2_PO_4_, 10 glucose, pH 7.35–7.45) at 37 °C and recovered for 30 min. The TG were then washed every 5 min for 30 min in total, followed by treatment with the following drugs for 20 min. Four series of experiments were designed. Series 1: In order to validate this TG tissue model for determining CGRP release, KCl, the known stimulus for neuronal activation and CGRP release [40], was applied. Two groups were designed: (i) Kreb’s (*n* = 8); (ii) 40 mM KCl (*n* = 8). Series 2: In order to determine whether SFKs activity participates in TRPA1-mediated CGRP release and IL-1β gene expression in TG, the effects of a SFKs inhibitor, saracatinib (S1006 Selleckchem, Houston, TX, USA), on their levels induced by a TRPA1 activator, umbellulone (SML0782 Sigma-Aldrich, St. Louis, MO, USA), were examined. In this series, three groups were designed: (i) vehicle (0.06% DMSO) (*n* = 8); (ii) 600 μM umbellulone (*n* = 8); (iii) 600 μM umbellulone + 1.5 μM saracatinib (*n* = 8). Series 3: In order to determine whether PKA activation promotes CGRP release and IL-1β gene expression in TG and whether SFKs activity regulate these processes, the effects of a PKA activator, dbcAMP (S7858 Selleckchem, Houston, TX, USA) alone, and co-treatment of dbcAMP and the SFK inhibitor saracatinib on CGRP release and IL-1β gene expression were examined. In this series, three groups were designed: (i) Kreb’s (*n* = 8); (ii) 300 μM dbcAMP (*n* = 8); (iii) 300 μM dbcAMP + 1.5 μM saracatinib (*n* = 8). For series 1, 2 and 3, TG tissue culture media and TG were collected for measuring CGRP level by Enzyme-linked Immunosorbent Assay (ELISA) and IL-1β mRNA level by Quantitative Polymerase Chain Reaction (qPCR), respectively. Series 4: In order to investigate whether SFKs are activated in response to TRPA1 activation and whether this process can be regulated by PKA in TG, the effects of umbellulone in the presence or absence of a PKA inhibitor, PKI (14–22) Amide (476485 Sigma-Aldrich, St. Louis, MO, USA), on phosphorylation of SFKs at Y416 were examined. Three groups were designed: (i) vehicle (0.06% DMSO) (*n* = 8); (ii) 600 μM umbellulone (*n* = 8); (iii) 600 μM umbellulone + 10 μM PKI (14–22) Amide (*n* = 8). In this series, TG were collected for extracting total proteins and measuring the level of phosphorylated SFKs using WB as described above.

### 4.6. ELISA

The level of CGRP released into mouse TG culture media was measured by using a CGRP ELISA Kit (CSB-EQ027706MO, CUSABIO, Houston, TX, USA). Briefly, tissue culture media was added into an assay plate precoated with CGRP antibody followed by incubating with biotin-conjugated antibody specific for CGRP and subsequently with avidin-conjugated Horseradish Peroxidase. The wells were added with TMB substrate for developing color proportional to the amount of CGRP from tissue culture media bound to the CGRP antibody precoated in the wells. The optical density was read at 450 nm, 540 mm and 570 nm, respectively using a microplate reader (BioTek). A standard curve relating optical density to concentration of CGRP in standard solutions was plotted and used to determine the concentration of CGRP.

### 4.7. qPCR

Total RNA of mouse TG was extracted using TRIZOL reagent (T9424 Sigma-Aldrich, St. Louis, MO, USA) and was reverse transcribed to cDNA by a GoScript Reverse Transcription System (A5001 Promega, Madison, WI, USA). The level of IL-1β mRNA in mouse TG was detected by qPCR using GoTaq qPCR Master Mix (A6002 Promega, Madison, WI, USA). Two housekeeping genes, peptidylprolyl isomerase A (PPIA) and β-actin (ACTB), were used as reference genes. Primers specific to the targeted genes were as follows: IL-1β forward 5′ACTACAGGCTCCGAGATGAACAAC3′, reverse 5′CCCAAGGCCACAGGTATTTT3′; ACTB forward 5′CTGTCCACCTTCCAGCAGAT3′, reverse 5′CGCAGCTCAGTAACAGTCCG3′; PPIA forward, 5′TTGCTGCAGACATGGTCAAC3′, reverse 5′TGTCTGCAAACAGCTCGAAG3′. The qPCR reaction was performed in QuantStudio 5 Real-Time PCR System (Applied Biosystems, Waltham, MA, USA). The level of individual IL-1β mRNA was normalized to the geometric mean of the mRNA levels of the 2 reference genes.

### 4.8. Co-Immunoprecipitation (Co-IP)

The physical interaction between TRPA1 and SFKs in rat cerebral cortex (*n* = 1) was detected by Co-IP using a Pierce Classic IP Kit (26146 Thermo Scientific, Waltham, MA, USA) and WB. Briefly, 1 mg of protein was incubated with 8 μg of the anti-TRPA1 antibody, 1 μg of the anti-SFKs antibody (2109 CST, Beverly, MA, USA) or IgG (D110502 BBI Life Sciences, Shanghai, China) at 4 °C overnight, followed by incubating immune complex with A/G protein agarose resin at 4 °C for 1 h. The captured immune complex was eluted and collected from the resin for WB using the anti-SFKs antibody (1:400) for detecting the SFKs and the anti-TRPA1 antibody (1:100) for detecting TRPA1.

### 4.9. Statistical Analysis

Data were analyzed using GraphPad Prism. Normality test was performed for all quantitative data by Shapiro–Wilk test. For comparisons between two independent groups, if the data passed the normality test, the data were presented as mean ± standard error of the mean and analyzed by a two-tailed unpaired *t*-test; if not, the data were presented as median (interquartile range) and analyzed by a Mann–Whitney test. Two-tailed Pearson correlation was used for correlation analysis between two parameters. The degree of correlation was shown by the correlation coefficient r (−1 ≤ r ≥ +1). Significant differences were shown by * *p* < 0.05, ** *p* < 0.01, *** *p* < 0.001, or **** *p* < 0.0001.

## Data Availability

Not applicable.

## Supplementary Materials

The following are available online at https://www.mdpi.com/article/10.3390/ijms222212273/s1, Figure S1: Anti-TRPA1 antibody did not reduce phosphorylation of SFK at Y416 induced by multiple CSD in membranes of rat cerebral cortices.
