# Supplementary material for: TRPA1-Mediated Src Family Kinases Activity Facilitates Cortical Spreading Depression Susceptibility and Trigeminovascular System Sensitization
Source: Int J Mol Sci. 2021 Nov 12;22(22):12273. doi: 10.3390/ijms222212273 (PMC8620265; doi:10.3390/ijms222212273)

Supplementary Figure S1. Anti-TRPA1 antibody did not reduce phosphorylation of SFK at Y416 induced by multiple CSD in membranes of rat cerebral cortices.

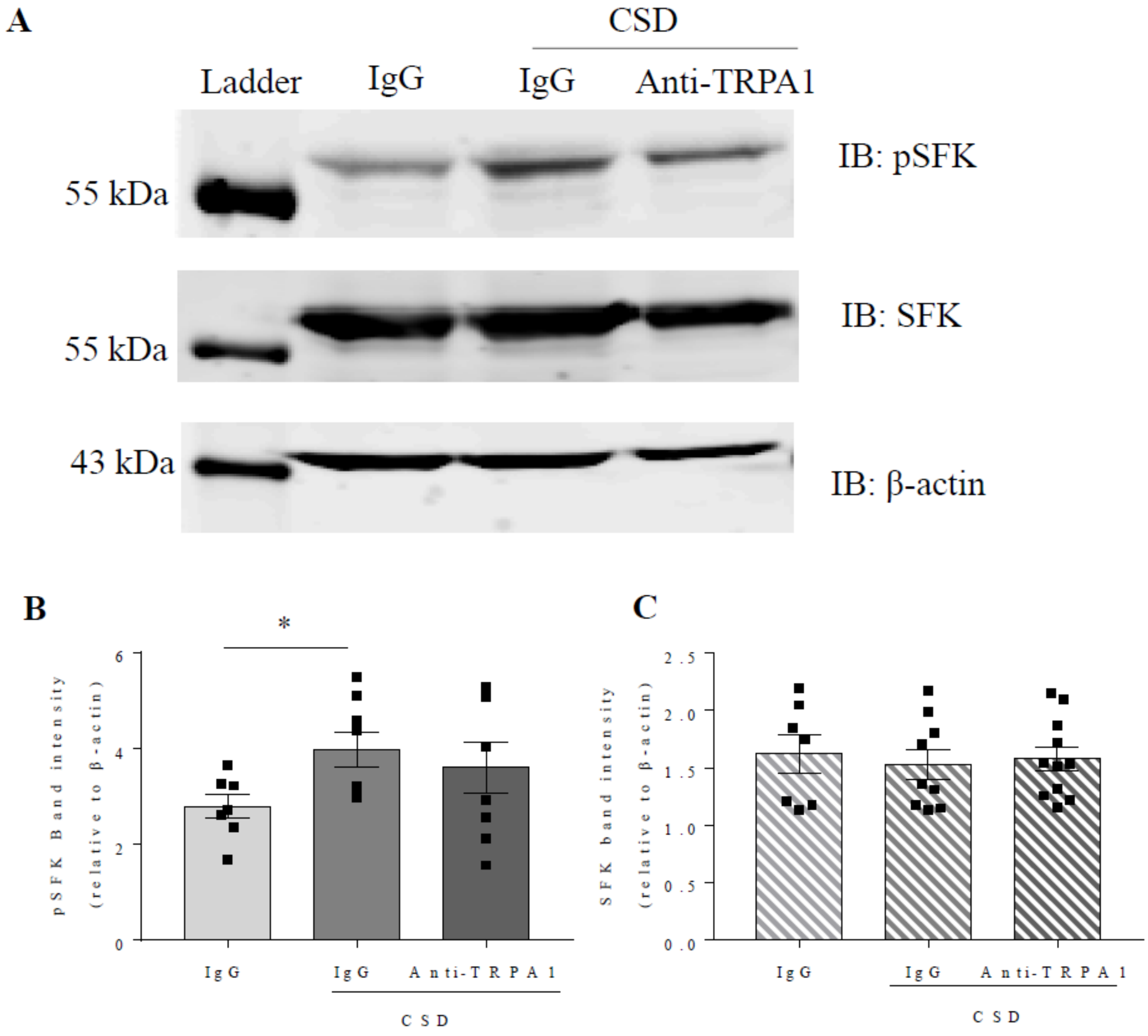

(**A**) Representative images showing western blot analysis of expression of phosphorylated SFK (pSFK) at Y416, SFK and β-actin in membrane cerebral cortices of rats treated with IgG or anti-TRPA1 antibody (Anti-TRPA1) at 0.8 μg with or without 2 M K^+-induced CSD. (**B**,**C**) Data analysis of the expression levels of phosphorylated SFK at Y416 or SFK relative to that of β-actin in membrane cerebral cortices of rats treated with IgG without CSD induction (n = 7), IgG with CSD induction (n = 8) or anti-TRPA1 antibody with CSD induction (n = 8). Two-tailed unpaired t test was used for comparison between IgG without CSD and IgG with CSD group, IgG with CSD and anti-TRPA1 antibody with CSD group. CSD augmented the level of relative band intensity of phosphorylated SFK at Y416 to 3.97 ± 0.37 (n = 8) in membrane of rat cerebral cortices in contrast to that at 2.78 ± 0.25 without CSD induction ($n = 7$, $p = 0.0201$)

Supplementary Figure S2. Original blots for the representative images presented in Figure 2.

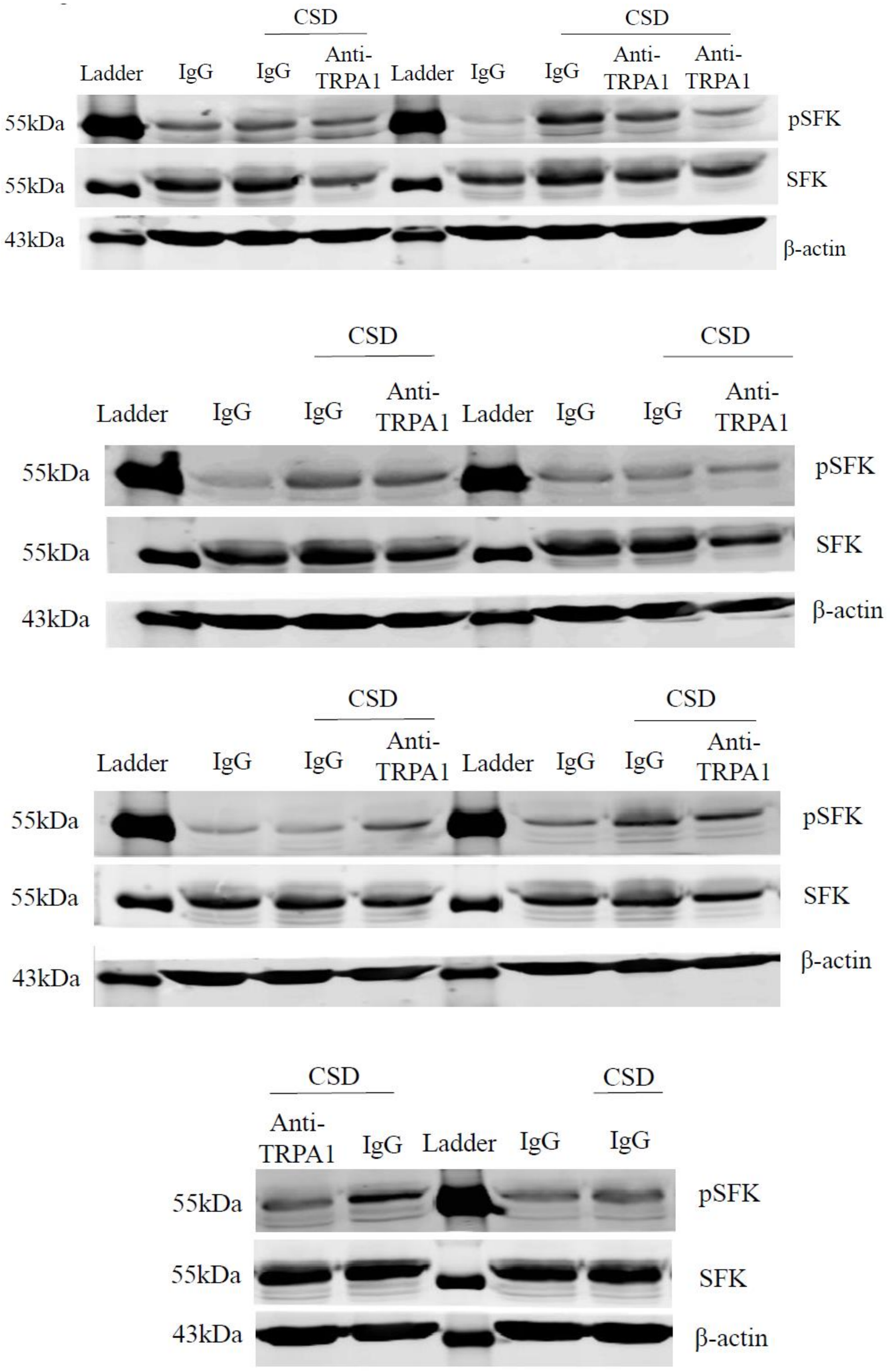

Supplementary Figure S3. Original blots for the representative images presented in Supplementary Figure S1

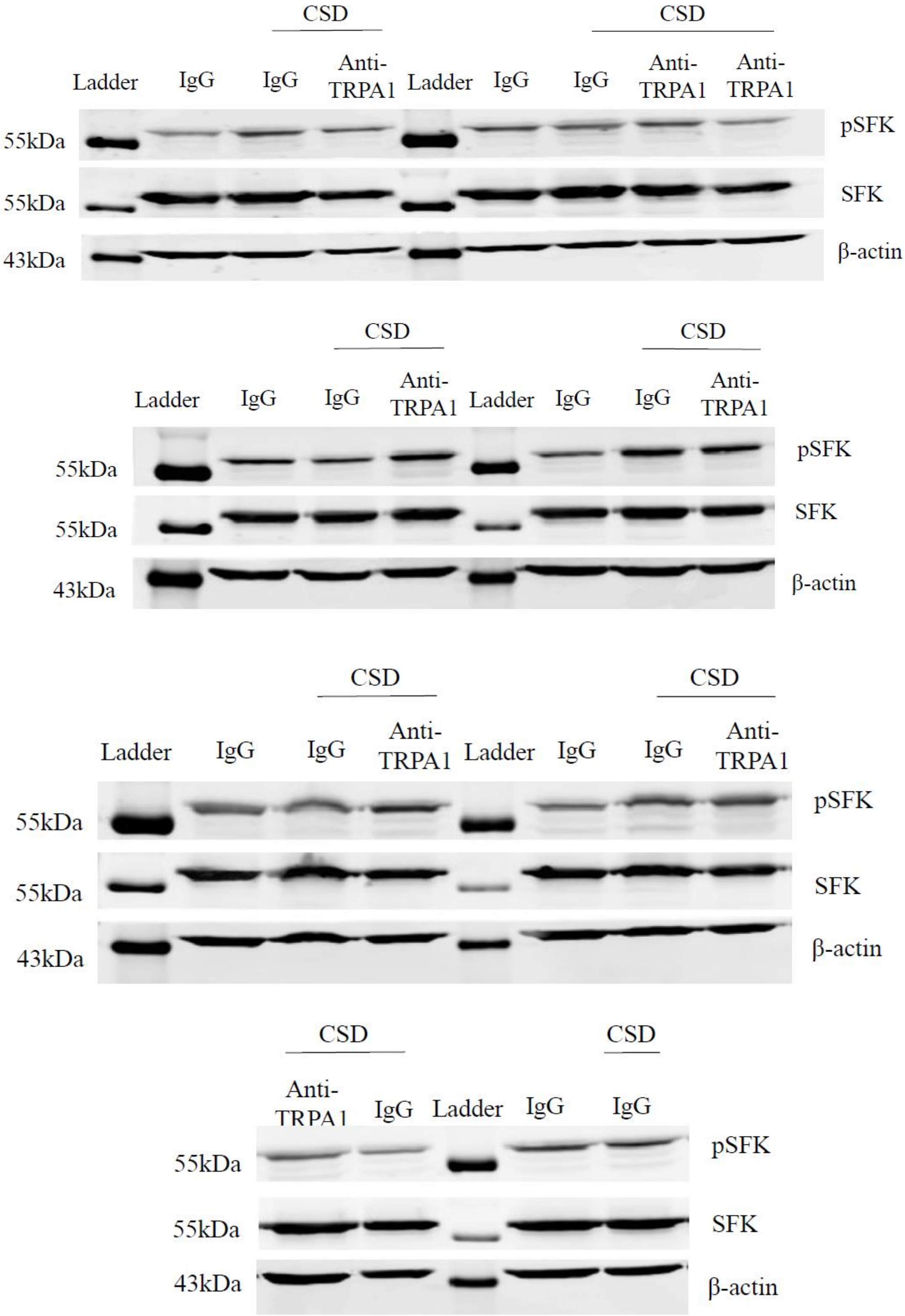

Supplementary Figure S4. Original blots for the representative images presented in Figure 5.

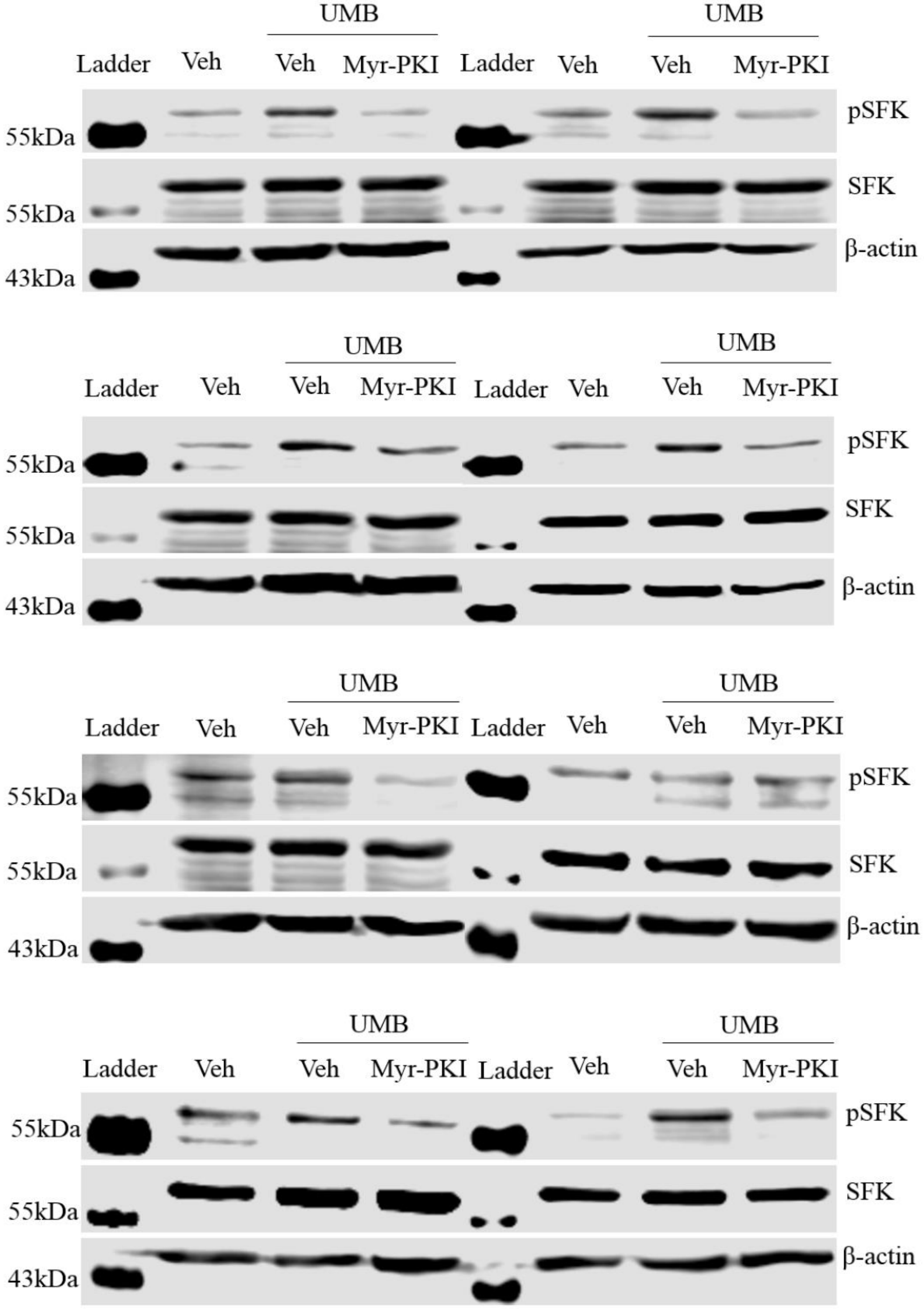

Supplement: Supplementary file 1 [file ijms-22-12273-s001.zip › ijms-1417524-SI.pdf]
